# A multimodal investigation of a pink-discoloured canine tooth in a jaguar (*Panthera onca*): a clinical, computed tomographic, microstructural, ultrastructural, and computer-aided design/ computer-aided manufacturing prosthodontic reconstruction study

**DOI:** 10.3389/fvets.2025.1674207

**Published:** 2025-10-24

**Authors:** Raluca Ioana Nedelea, Adrian Florin Gal, Vasile Rus, Sorin Marian Marza, Septimiu Tripon, Georgiana Deak, Mihai Marian Borzan, Gabriel Chișamera, Ovidiu Mureșan, Răzvan Vicențiu Dumitru, Cristinel Cezar Mătură, Paul-Stefan Panaitescu, Ioan Marcus

**Affiliations:** ^1^Universitatea de Stiinte Agricole si Medicina Veterinara Cluj-Napoca, Cluj-Napoca, Romania; ^2^Electron Microscopy Center “Prof. C. Craciun”, Faculty of Biology & Geology, “Babes-Bolyai” University, Cluj-Napoca, Romania; ^3^Muzeul National de Istorie Naturala Grigore Antipa, Bucharest, Romania; ^4^Private Practitioner, Cluj-Napoca, Romania; ^5^Dental Technician, Cluj-Napoca, Romania; ^6^Divet Medic All, Bucharest, Romania

**Keywords:** pink-discoloured tooth, jaguar, dental pulp, computed tomography, electron microscopy, CAD/CAM

## Abstract

Wild animals in captivity are prone to developing dental diseases. Pink-discoloured canine teeth in jaguars are often seen in wildlife photographs but are rarely reported in the literature, and none have been formally investigated. Within 24 h post-mortem, the oral cavity of a zoo jaguar was investigated using computed tomography (CT). One pink-discoloured canine tooth was atraumatically extracted, fixed, and stained for histological and transmission electron microscopy (TEM) examination. The intravitam pink-discoloured canine tooth exhibited no evidence of periodontal or periapical lesions. Microscopically, the dental pulp revealed numerous ectatic blood vessels with numerous thrombi that occluded the blood vessels. A high percentage of thrombi presented with the retunnelling phenomenon. Fluorescence imaging confirmed the presence of haemoglobin in the dentinal tubules. The study, the first of its type, sheds light on an intravitam pink-discoloured canine tooth opening, a hitherto unexplored topic in zoo dentistry. For the skull to be accepted into the zoological collection of the National Institute of Biology, the extracted canine tooth had to be replaced by a 1:1 scale prosthodontic reconstruction, macroscopically identical to the natural tooth. Prosthodontic reconstruction was performed using computer-aided design and computer-aided manufacturing (CAD/CAM) technology. This study, the first of its kind, investigates an intravitam pink-discoloured canine tooth in a jaguar—a hitherto unexplored topic in zoo dentistry—and describes its prosthodontic reconstruction.

## 1 Introduction

The jaguar (*Panthera onca*) (1) belongs to the Order Carnivora, Family Felidae, and Subfamily Pantherinae (2). As apex predators (3), jaguars have a unique, distinctive killing method that involves biting the skull (4–7) or cervical vertebrae (8, 9) to damage the central nervous system and render the prey unable to defend itself (10). To kill their prey, the jaguar penetrates the temporal bones, crushes, and removes the calvaria. In order to do this, jaguars have the following adaptations for durophagy: the skull is large and robust compared to the jaguar’s body size (7, 10). The canine teeth are large and robust but flattened (7) with a cylindrical aspect in the horizontal section (11, 12), and highly developed masseter and temporalis muscles (12). The canine teeth of jaguars can fulfil this function because they can withstand a bite force of 591 kg (1,399 lbs.) and are the third strongest among felids (7).

Oral pathologies are common in mammals, including jaguars (13). However, there is a paucity of information regarding intravitam pink discolouration of the teeth that has anecdotally been observed and documented in wildlife photography in jaguars. The presence and prevalence of oral pathologies in jaguars have been studied in retrospective studies (14, 15), cross-sectional ones (16–19), and case report series (13, 20). In these studies, only one tooth is reported to have a pink discolouration, without any associated radiographic pathology (19). Studies on post-mortem pink teeth in humans and animals have been published (21–24). Only one article (15) has reported post-mortem pink-discoloured teeth in jaguar skulls. Several figures in previously published articles (13, 15, 18–20) depict orange-pink discolourations of the canine teeth in jaguars that were not commented on by the authors.

Jaguar dentition is *diphyodont*, meaning that it has a deciduous and permanent set of teeth during its lifetime; *heterodont*, with different types of teeth; *secodont*, with sharp edges of the teeth and posterior teeth; and *brachydont*, involving short crowns with well-developed roots. The permanent dental formula is I3/3, C1/1, P3/2, and M1/1, totalling 30 teeth (2, 7).

Studies on the radiographic, histological, and ultrastructural characteristics of jaguar teeth are lacking within the existing literature. Our original research aims to present an investigation of a pink canine tooth in a jaguar, based on clinical findings, computed tomographic scanning of the head, microstructural and ultrastructural findings in the soft and hard tissues of the affected tooth, and electronic microscopic aspects of the coloured dentin, and to correlate these findings with those of previous studies. Finally, we detail the process of a CAD/CAM prosthodontic reconstruction of the tooth that was necessary to restore the skull’s natural appearance for a zoological collection.

## 2 Materials and methods

An approximately 12-year-old male jaguar (*Panthera onca*), weighing 39 kg, was found dead at the zoo. All four canine teeth exhibited pink discolouration, a condition that was first observed by keepers and reported to the attending veterinarian several years earlier. Because comparable discolouration had been noted in other individuals and no signs of oral pain were evident, no further diagnostic work-up was undertaken at that time. The carcass was maintained at 4 °C (and within 24 h post-mortem, the head was collected for analysis and preparation for the zoological collection). The oral cavity was examined macroscopically using a periodontal probe, followed by computed tomography (CT) imaging. Spiral CT scanning of the head and spine was performed using a Siemens CT Somatom Scope machine with 16 channels. The scan was performed with the skull in the dorsal recumbency. Head images were obtained in the axial plane using a 512 × 512 matrix, narrow windows (WW: 120, WL: 40), 3 mm slice thickness, and a pitch of 1.5. Multiplanar image reconstruction of the head was performed using soft tissue and bone window reconstructions at a slice thickness of 0.75 mm.

One pink-discoloured canine tooth (104) was extracted after obtaining consent from the Institute of Biology of the National Academy. The only requirement of the institution was for the tooth to be extracted without damaging the remainder of the skull. The surgical approach was made using a piezoelectric surgery unit, straight elevators, and dental forceps adapted to the dimensions of the tooth. Sharp dissection was performed using a number 15 scalpel blade to approach the maxillary bone, which was expanded slowly, and the periodontal ligaments were severed using piezoelectric equipment and dental elevators. Due to the relatively short time between the jaguar’s death and tooth extraction, a degree of bone elasticity was still present, which contributed to minimal skull damage during the procedure. The extracted tooth was transversely sectioned into two parts in the coronal part of the root. The section was performed with a high-speed handpiece and fissure tungsten carbide bur under cooling with saline solution to prevent pulp heating and alteration of pulpal proteins. The sectioned tooth was immediately immersed in 10% buffered formalin solution. At the end of the fixation period, the dental pulp was processed histologically by embedding it in paraffin, and the sections were stained using Goldner’s trichrome method. The dental hard tissues were decalcified with 12% trichloroacetic acid. Subsequently, some fragments were processed for light microscopy examination by embedding the sample in paraffin and sectioning into 5-μm-thick histological sections using a Leica rotary microtome (Model RM2125, Leica Biosystems, Nussloch, Germany). The achieved tissue sections were stained using Goldner’s trichrome (GT) method and assessed using an Olympus BX41 light microscope (Olympus Corporation, Tokyo, Japan). Microphotographs were obtained using an Olympus SC180 Microscope Digital Camera (Olympus Corporation, Tokyo, Japan) along with Olympus cellSens Entry 3.1 software (Olympus Corporation, Tokyo, Japan). Optical fluorescence microscopic imaging was performed on the same tissue samples using a Zeiss Axio Lab A2 microscope. A 100×/1.25 oil objective was employed, with excitation provided by a 470 nm LED, a 515 nm long-pass emission filter, and an Erc5s camera for image acquisition.

The remaining tissue samples were processed for transmission electron microscopy (TEM). These samples were pre-fixed with 2.7% glutaraldehyde in 0.1 molar (M) phosphate buffer for 2 h at 4 °C. Later, the samples were washed in four baths, each for 1 h, with 0.15 M phosphate buffer (pH 7.2). They were then post-fixed with 2% osmium tetroxide in 0.15 M phosphate buffer for 1 h and 30 min at 4 °C. At the end of the fixation process for TEM, the samples were dehydrated with acetone and embedded in an epoxy resin (Epon 812). The blocks were cut using an ultramicrotome at a thickness of 80 nm (Leica UC 6). The sections performed were contrasted with UranyLess and lead citrate solutions, examined under the electron microscope (Jeol JEM1010), and photographed.

A virtual simulation software (Exocad) enabled the creation of a three-dimensional (3D) digital model based on CT scan images. To create a macroscopically identical tooth, a metallo-ceramic prosthodontic crown was computer-aided designed. CT images provided a 3D volumetric dataset in a standard medical imaging format, Digital Imaging and Communications in Medicine (DICOM). This dataset was imported into Exocad, a specialized dental CAD software. Using segmentation tools, the technician isolated the tooth. After obtaining the virtual model, the technician designed the titanium core of the future canine tooth, with a 2 mm reduction in diameter and a 4 mm reduction in length, to leave a 2 mm width of space for ceramic application around the tooth to obtain aesthetics. In Exocad, additional fine adjustment can be done to ensure margins, occlusion, and functionality. The final design was exported as a stereolithography (STL) file to a CAD module to 3D print the titanium core. As the CAD module was designed for human dentition and the length of the jaguar’s canine tooth was longer than any human tooth, it was necessary to divide the core into three segments. The metal was sandblasted to roughen the surface and improve the bonding with the ceramics. A thin layer of opaque porcelain was applied and fired in a porcelain furnace at approximately 1,000 °C. In addition, the technician applied dentin porcelain and an enamel layer of ceramic, firing the tooth again in the furnace after each layer to sinter the ceramic particles. Shades of pink had to be added to obtain a similar colour to the original tooth. Finally, an external layer of glaze was applied, resulting in a shiny and smooth surface.

## 3 Results

### 3.1 Clinical results

**Figure 1:**
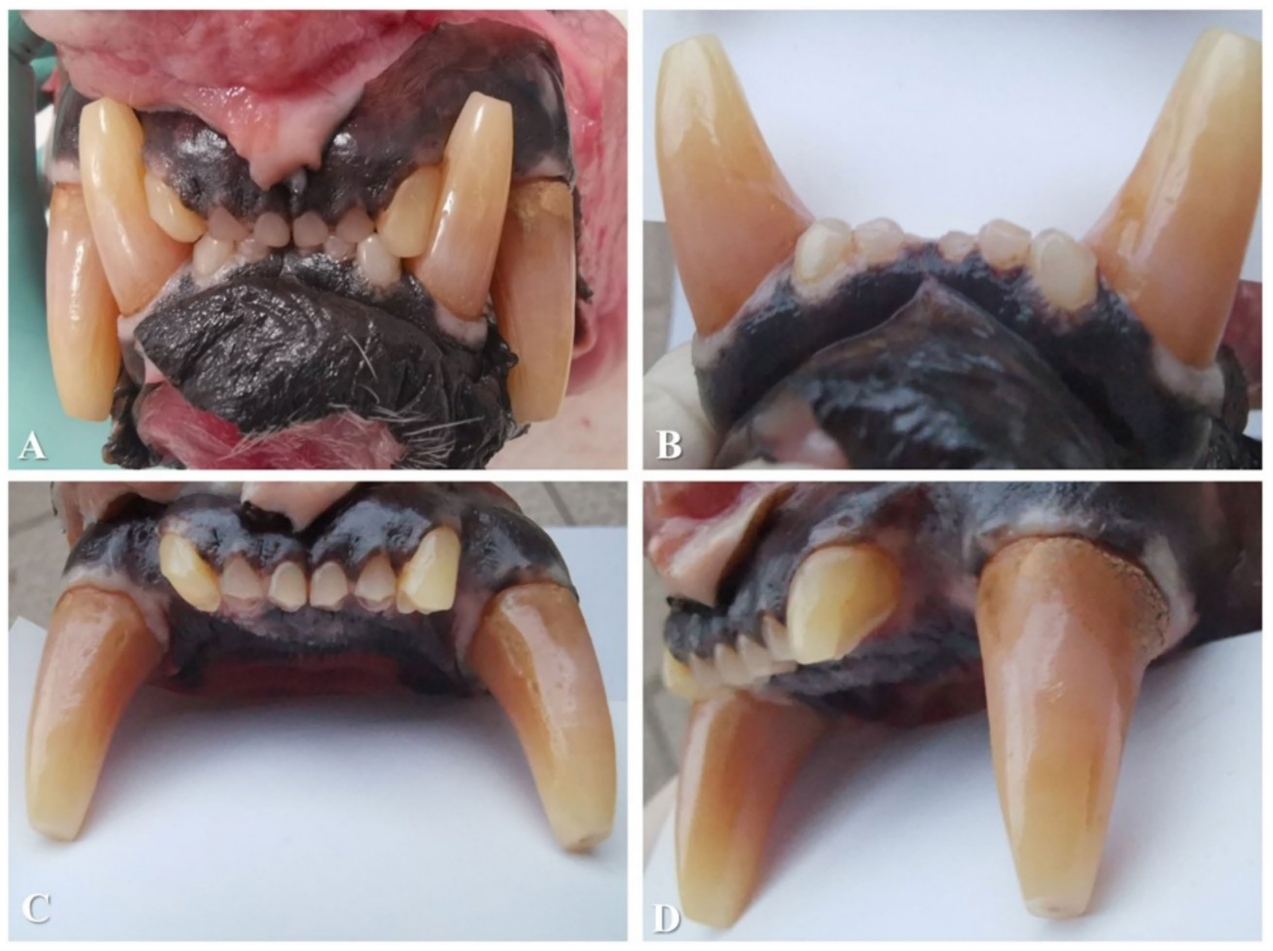
**(A)** Rostral view of the bite. Note the occlusal edge contact between the incisor teeth, the orange-pink colour of the canine teeth, and dental attrition. The pink appearance visible in some incisors in these images resulted from illumination and not from true intravitam discolouration of the dental tissue. **(B)** Buccal view of the mandibular incisor and canine teeth. Note the normal colour of the incisor teeth compared to a blank page that is positioned caudally to them for comparison. **(C)** Buccal view of the maxillary incisor and canine teeth. **(D)** View from the left maxillary incisor and canine teeth showing normal colouration of the incisor teeth.

A class 1 malocclusion, characterized by rostral crowding and tooth-on-tooth malocclusive contact between 103 and 404 and between 203 and 304, with a level rostral bite, was found (Figure 1).

The 401 was clinically absent from the dental arch. All four canine teeth presented with superficial abrasion of the cusps of the crowns and pink-orange crown discolouration. Because of the occlusal edge contact between the incisor teeth, all incisors presented with attrition of the crowns with wear facets on the incisal edges. None of these lesions extended into the pulp. The close contact between 103 and 404 and between 203 and 304 caused attrition on the mesial sides of 304 and 404 and on the distal sides of 103 and 203. 103 had an uncomplicated crown fracture at the disto-incisal angle of the crown.

Deposits of calculus were present on the buccal sides of the maxillary premolar teeth. The 104, 107, 204, and 207 each had 4 mm of buccal calculus coverage, measured from the gingival margin towards the cusp. The 108 and 208 each had the entire buccal side covered with calculus. As the examination was conducted post-mortem, no gingival bleeding could be observed, but no overt inflammation of the sulcular or other gingival tissues was noted. No other signs of periodontal disease were found. No abnormalities were identified in the soft tissues of the oral cavity. The extracted canine tooth, 104, measured 75 mm in total length with a crown length of 27 mm, as measured from the level of the cementoenamel junction to the cusp of the tooth post-extraction.

### 3.2 Computed tomographic results

The images from the sagittal view revealed the midline structure of the skull, an elongated nasal cavity, the maxilla with the canine teeth, and the occipital region. The sagittal plane highlighted the alignment of the cranial vault and the mandibular symphysis. The dorsal view displayed a frontal section through the rostral portion of the skull, emphasizing the bilateral symmetry of the nasal passages, orbits, and maxillary sinuses. This view also captured the bone structure of the zygomatic arches and alignment of the maxillary teeth, including the canine and premolar teeth. The transverse view provided a cross-sectional perspective at the level of the orbits, revealing the orbital cavities, frontal bone, and maxillary region. This view underscored the thickness of the cranial bones and the spatial relationship between the orbits and the nasal cavity.

In the dorsal view, the maxillary teeth were partially visible through the nasal and orbital regions. The incisor teeth, positioned rostrally, were small and closely spaced. The canines extended ventrally with roots anchored in the maxilla. The premolar and molar teeth, although less visible in the dorsal view, were positioned caudally. The maxillary carnassial teeth (108 and 208) exhibited a sharp, blade-like morphology, typical of felids. The lateral view offered a clear profile of the dentition within the context of the skull’s overall structure. The incisors were small and aligned in a row, rostral to the canines. The canines were elongated, conical, and slightly curved, and the sharp edges of the premolar teeth, particularly 108 and 208, were clearly visualized. The dorsal view provided a detailed perspective of the mandibular dentition and palate. The mandibular incisor teeth were small and closely spaced. The mandibular canines mirrored the maxillary ones in size and shape, forming a powerful occlusal key. Caudally, the premolar and mandibular carnassial teeth (309 and 409, respectively) exhibited sharp cusps.

**Figure 2:**
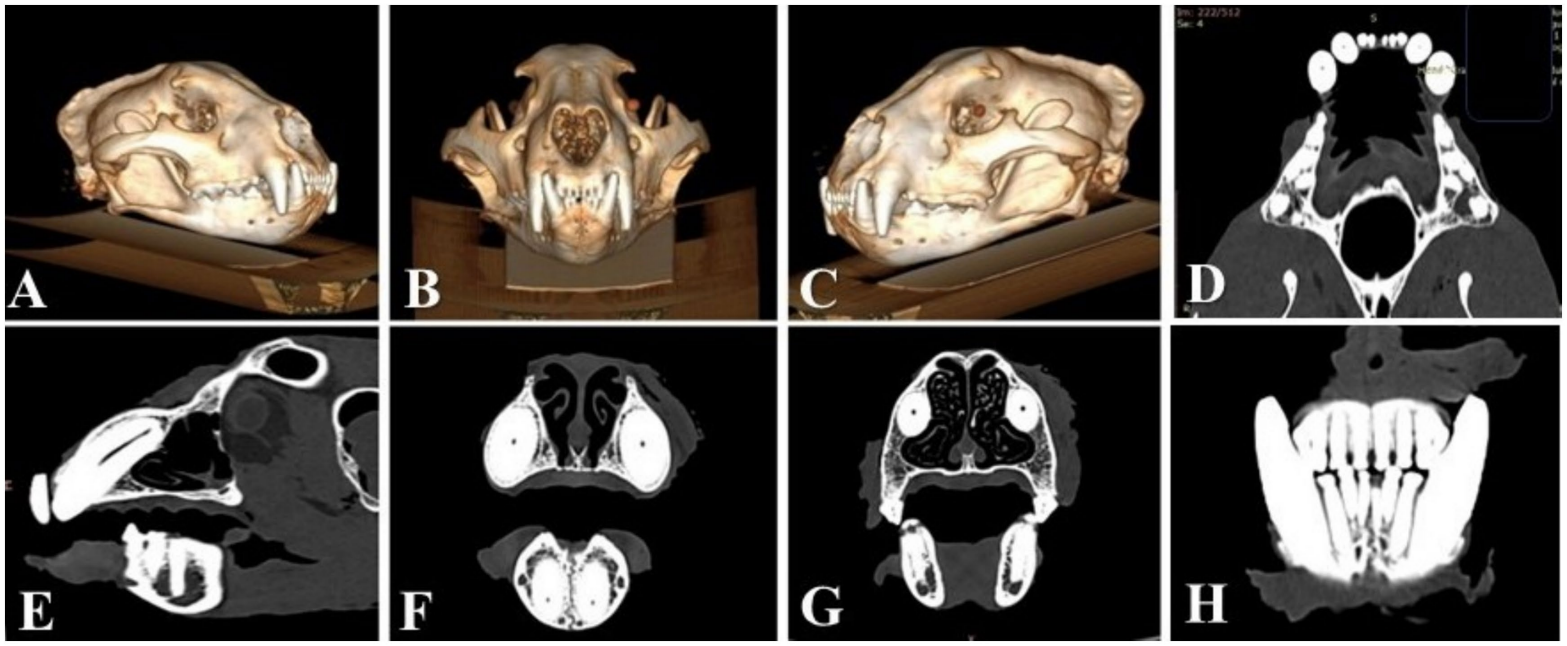
**(A)** 3D reconstruction of the skull—right lateral view of the skull. **(B)** 3D reconstruction of the skull—oro-aboral view of the skull. **(C)** 3D reconstruction of the skull—left lateral view of the skull. **(D)** Dorsal CT image of the bone window reveals similar dimensions of the pulp cavities in all four canine teeth. **(E)** Sagittal CT image of 104, revealing the pulp cavity with no periapical pathologies or bone window. **(F)** Transverse CT images of the four canines were used to compare the dimensions of the pulp cavities. All four canines have similar dimensions of the pulp cavities, with no abnormalities or bone windows. **(G)** Transverse CT image through the roots of the maxillary canine teeth for comparison, showing similarities in the dimensions of the pulp cavities and the bone window. **(H)** Transverse CT image that enables visualization of the 401 root remnant.

The pulp cavities of all canine teeth had no abnormalities and similar canal widths to one another. No signs of resorptive lesions or periodontal disease could be found on the CT scan. The temporomandibular joints had no alterations. Reconstruction of the skull based on the CT scan and details regarding the canine teeth is shown in Figure 2. The root of the 401 was present inside the mandibular bone (Figure 2H).

### 3.3 Microstructural and ultrastructural study results

**Figure 3:**
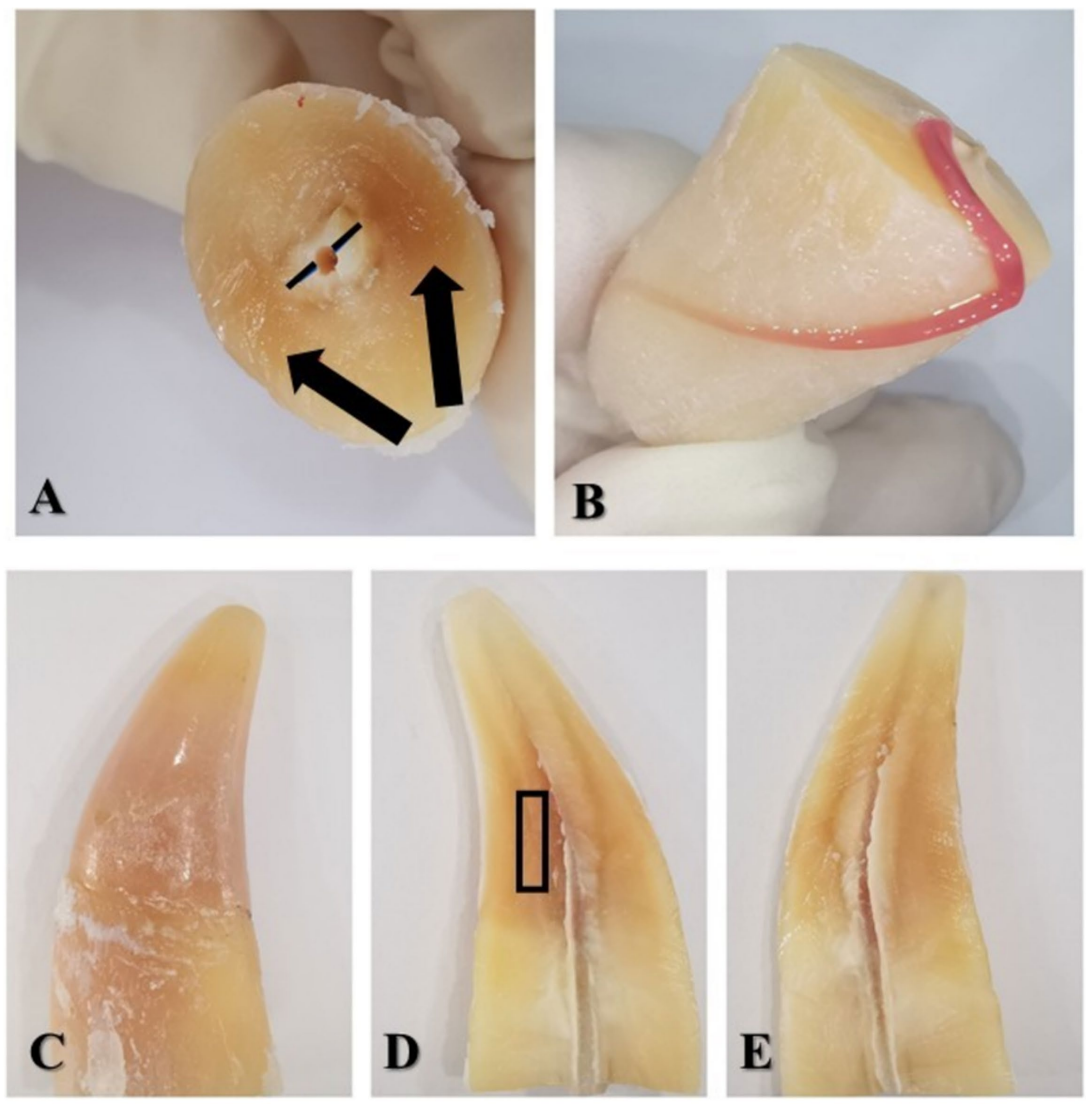
**(A)** Coronal part of the sectioned tooth—view from the cervical part; black lines—predentin zone; black arrows show the staining of the dentin. **(B)** The radicular part of the 104 with the coronal part of the dental pulp laid out on the root surface. **(C)** Coronal part of the sectioned tooth in buccal view. **(D)** Buccal half of the crown—the pulp chamber stained with dentin. The black rectangle shows the region of the dentin harvested for TEM. **(E)** Palatal half of the crown—the pulp chamber with dentin staining.

After the transverse section of the tooth, the predentin zone could be seen around the dental pulp (Figure 3A). The modified colour of the dentin was most prominent buccally and palatally. The soft tissue of the pulp was pink-red in colour without any signs of haemorrhage or necrosis.

**Figure 4:**
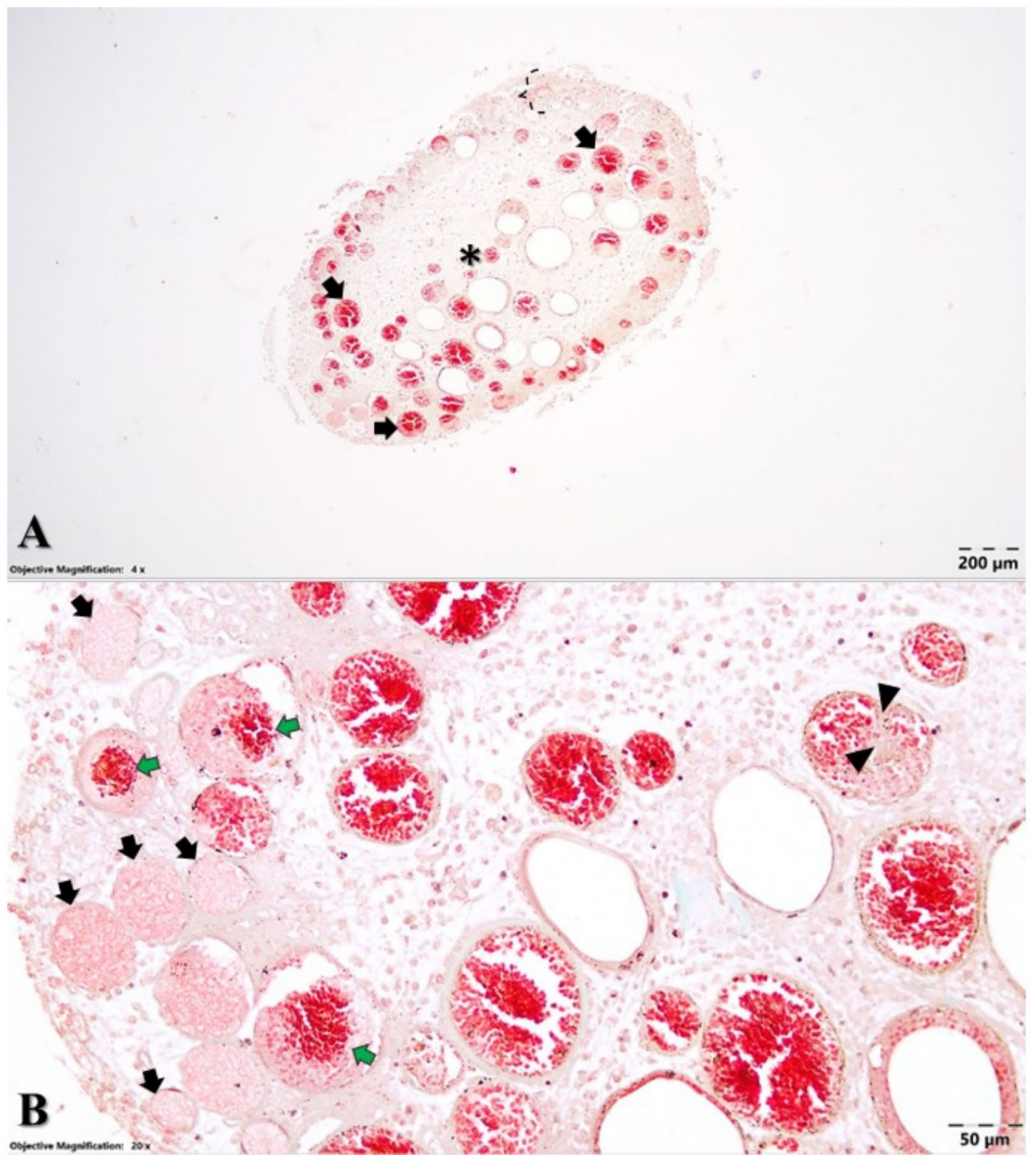
Microstructural aspects of the dental pulp **(A)** cell-rich zone (accolade) and central part of the coronal dental pulp (asterisk) with an oedematous aspect and numerous ectatic blood vessels (black arrows) and Goldner’s trichrome staining. **(B)** Dental pulp with numerous occluding fibrinous thrombi (black arrows), some of them showing recanalization (green arrows) and fibrous organization (arrowheads). Goldner’s trichrome staining.

The cell-rich zone and the central part of the coronal pulp were highly vascularized. Numerous blood vessels presented with occluding fibrinous thrombi, and some of them presented with recanalization and fibrous organization (Figure 4). Some neutrophils and siderophages have been found in small numbers. No bacteria were present.

**Figure 5:**
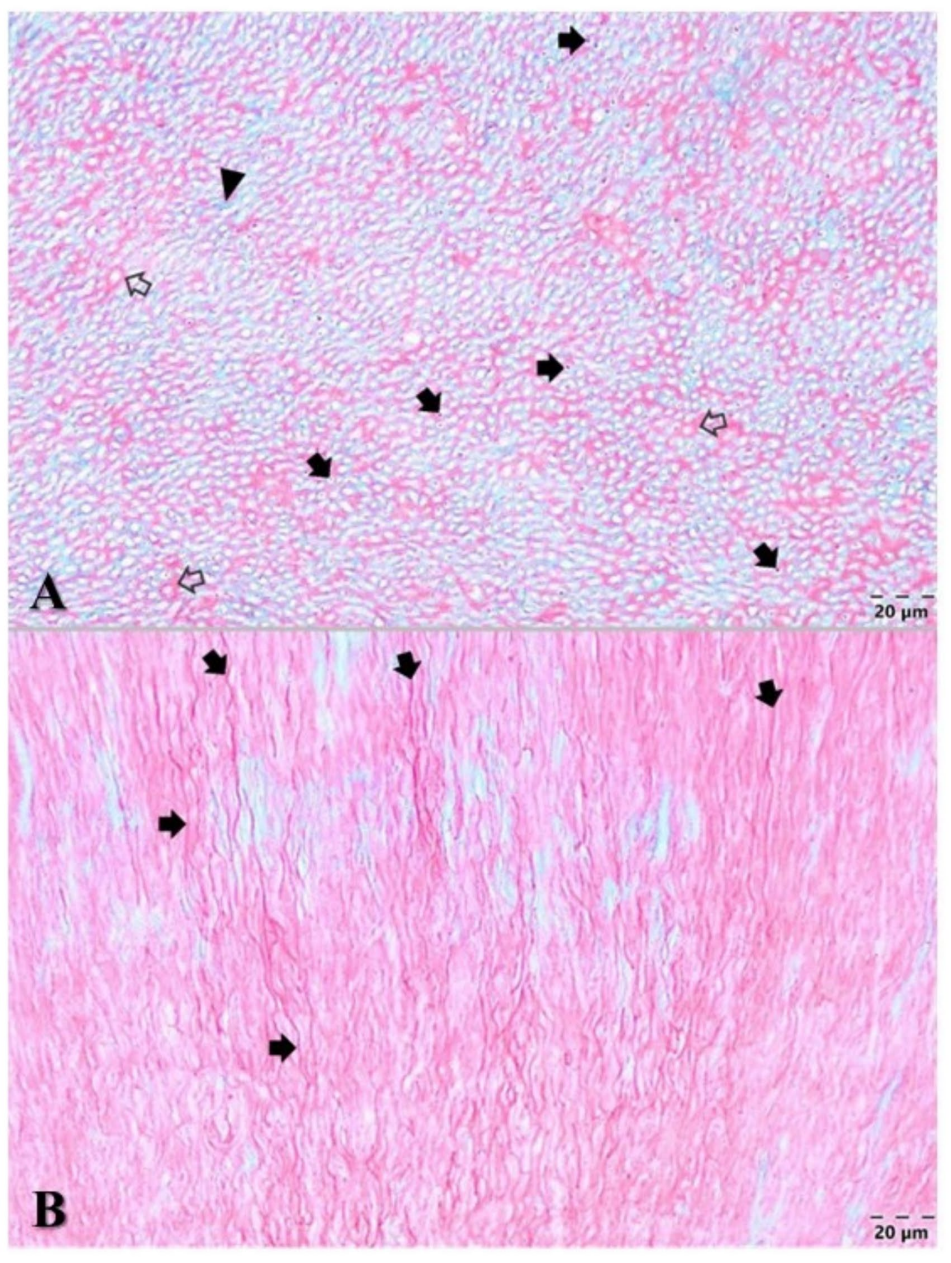
Microstructural aspects of the dentin. **(A)** Cross-section of dentinal tubules containing odontoblastic processes (black arrows), which are surrounded by the peritubular acidophilic dentin (blank arrows), while the intertubular dentin displayed a basophilic appearance (arrowhead) with Goldner’s trichrome stain. **(B)** Longitudinally sectioned oedematous odontoblastic processes inside the tubules (black arrows) of the dentin. Goldner’s trichrome stain.

Regarding the hard tissues of the assessed tooth, the dentin structure presented numerous dentinal tubules, most of them characterized by normal-sized or hypertrophied oedematous odontoblastic processes (Figure 5). The peritubular dentin that encircled each tubule had an acidophilic appearance, whereas the intertubular dentin displayed a basophilic appearance.

**Figure 6:**
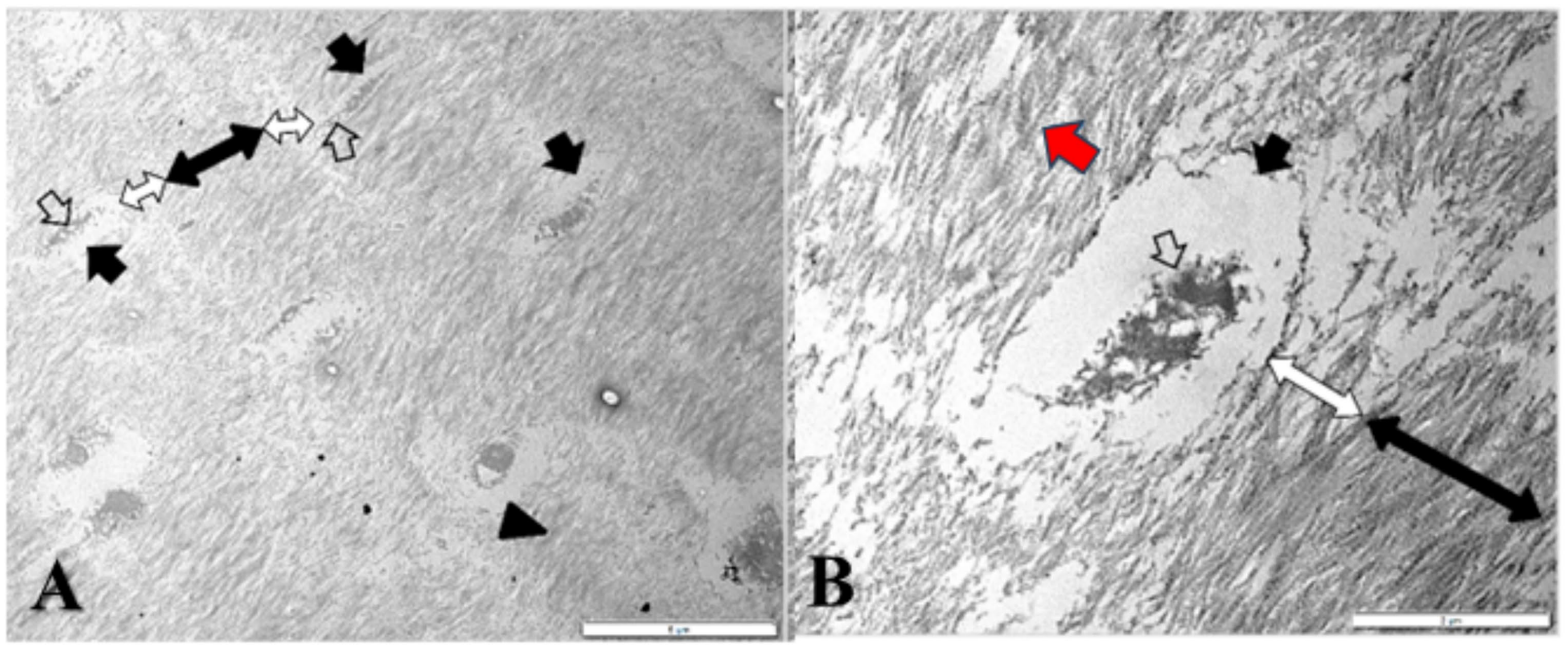
TEM **(A)** General view of the dentin and **(B)** Dentinal tubules (black arrows) investing vacuolated odontoblastic processes (white arrows); peritubular dentin (white double-ended arrows) and intertubular dentin (black double-ended arrows) with interlacing collagen bundles (arrowhead) showing a distinctive banding pattern (red arrow).

On the TEM images, the typical structure of dentin was observed, specifically dentinal tubules enclosing odontoblastic processes (Figure 6, white arrow). In the ultrastructure of dentin, collagen fibres showed a distinct appearance in the peritubular region compared to that in the intertubular region. In both dentinal zones, the orientation of the collagen fibres was three-dimensional (Figure 6, black double arrow end), with longitudinal, oblique, and transverse fibres intercepted in the TEM sections. In the peritubular region, the dentin exhibited a much lower density of collagen fibres that were occasionally organized in bundles (Figure 6, white double arrow end). However, in the intertubular dentin, the density of the collagen fibres was very high, and the vast majority of the fibres tended to be organized in small interlacing bundles, each of which showed a banding pattern. Additionally, the intertubular collagenous bundles were thicker compared to the peritubular ones.

**Figure 7:**
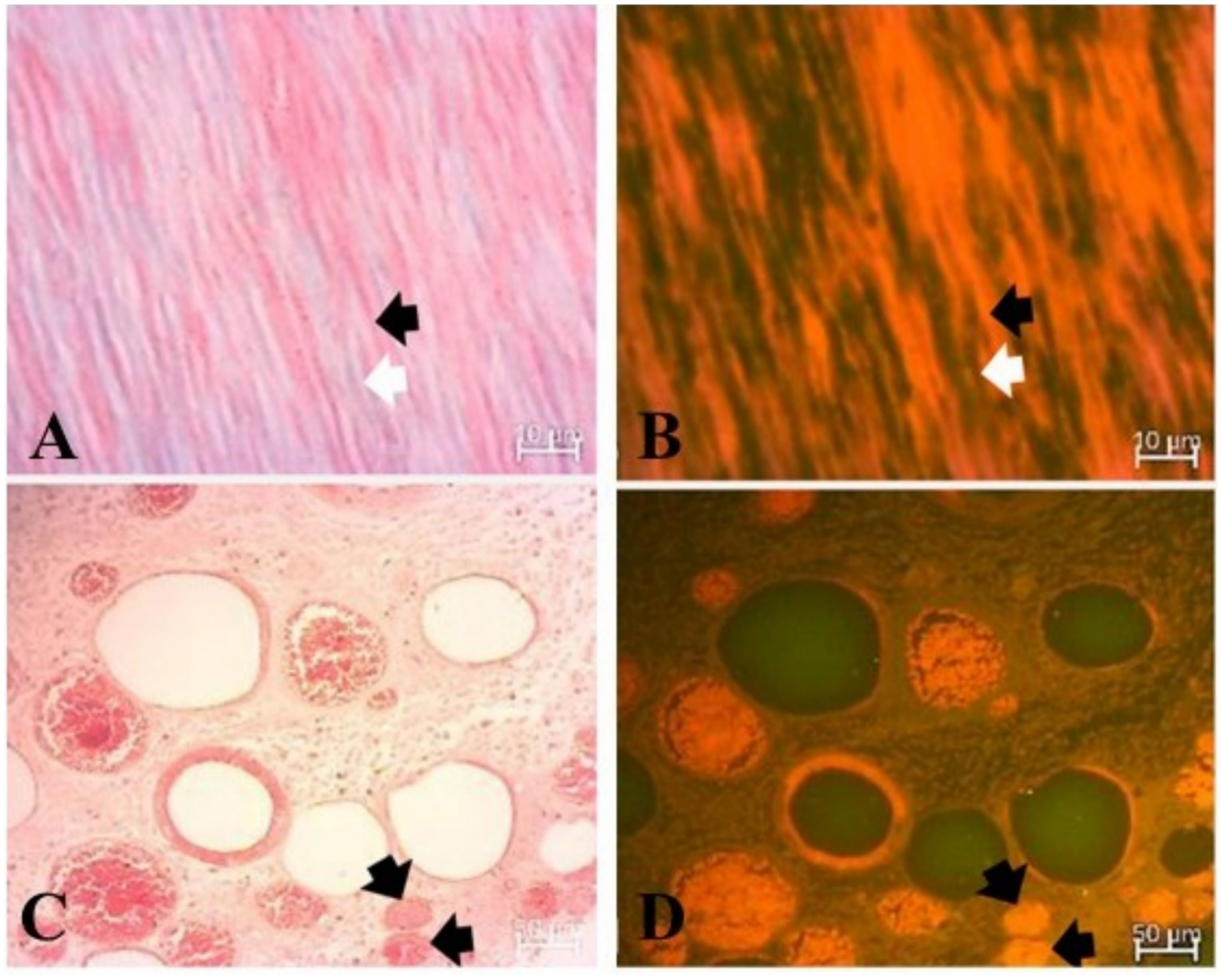
Fluorescence imaging of the dentin and dental pulp. **(A)** Longitudinally sectioned dentinal tubules (white arrows) in optical microscopic appearance, dentin flanking the tubules (black arrow), and Goldner’s trichrome (GT) stain. **(B)** Longitudinally sectioned dentinal tubules (white arrow) in optical fluorescence imaging with a 470 nm LED for excitation and a 515 nm long-pass emission filter. Note the fluorescence of the dentin due to infiltration with haemoglobin (black arrow). **(C)** Dental pulp with occluding fibrinous thrombi (black arrows) in optical microscopic appearance. **(D)** Dental pulp with occluding fibrinous thrombi in optical fluorescence imaging with a 470 nm LED for excitation, confirming haemoglobin-rich fluorescence (black arrows).

Optical fluorescence imaging (Figure 7) confirmed the haemoglobin’s autofluorescence (Figure 7D) as it was easily identified in thrombi. Moreover, the fluorescence optical imaging identified haemoglobin in the dentinal tubules (Figure 7B).

**Figure 8:**
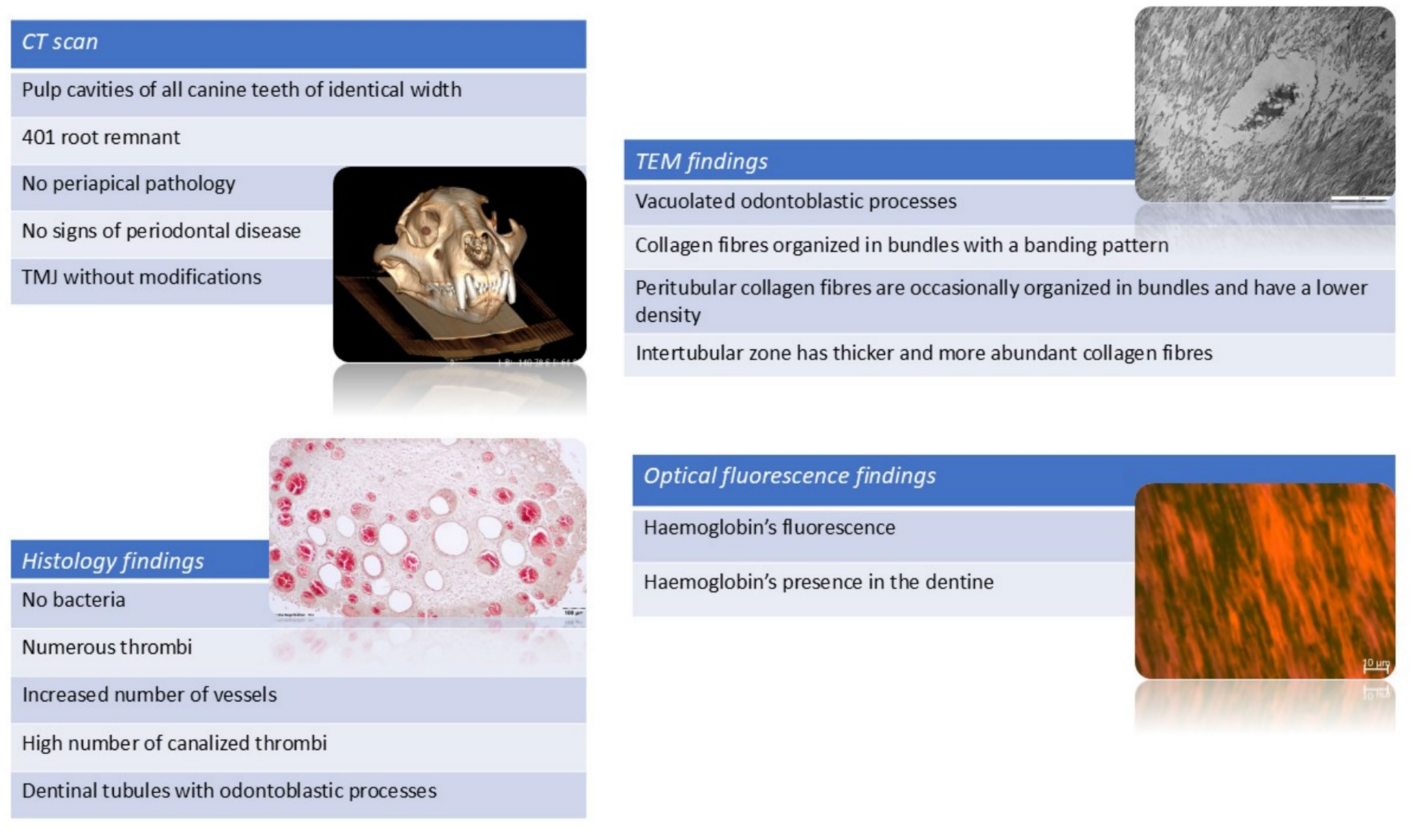
Summary of results from all assessment modalities: computed tomography, histology, transmission electron microscopy, and optical fluorescence imaging.

The results of CT scan, histology, electron microscopy and optical fluorescence are summarised in a graphic way, in Figure 8.

### 3.4 CAD/CAM prosthodontic reconstruction results

**Figure 9:**
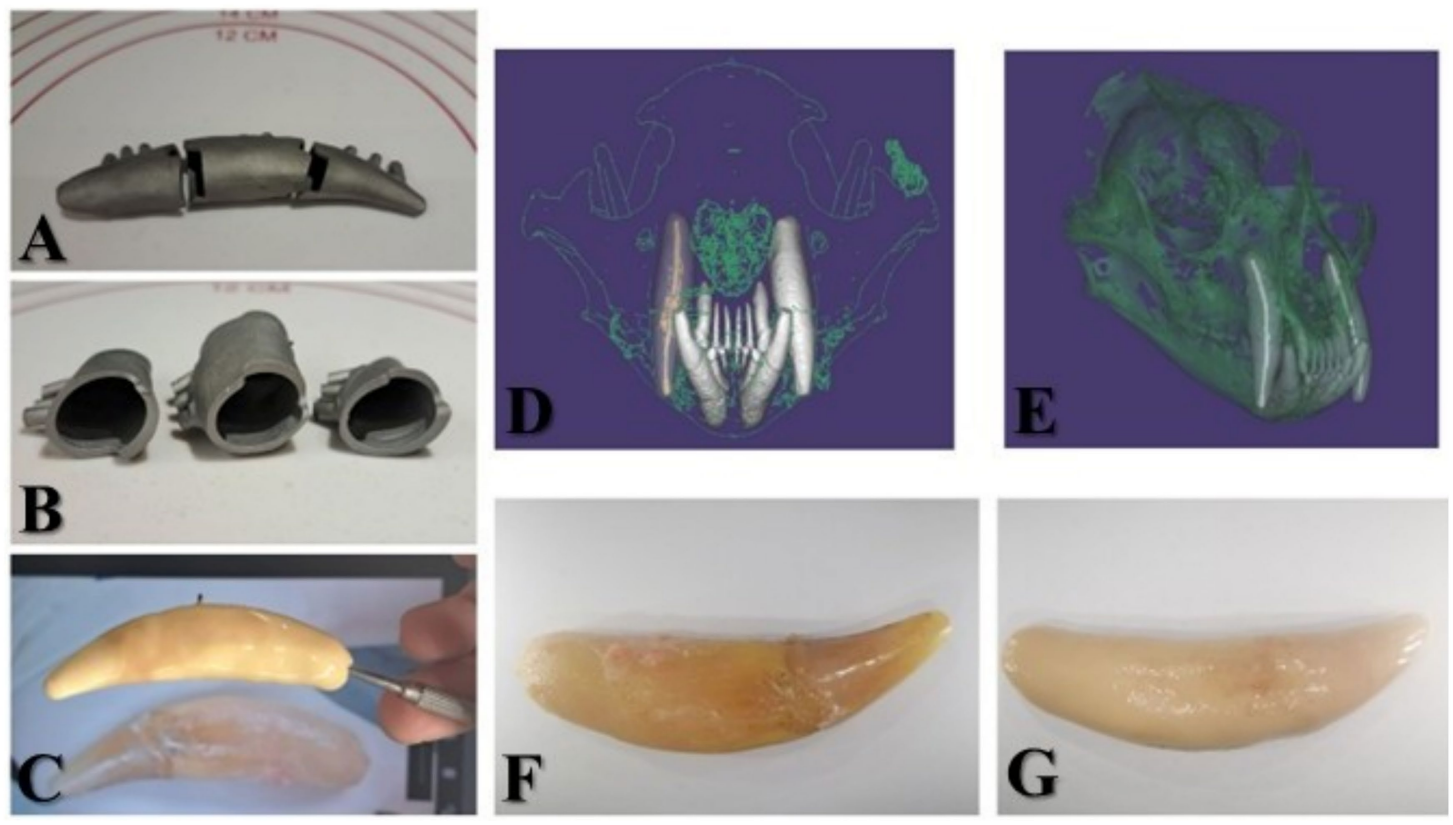
**(A)** The three-part titanium-printed core of the prosthodontic reconstruction. **(B)** Interior view of the titanium base. **(C)** Applying the porcelain lining and comparing it with the original image of 104. **(D)** Oro-aboral view of skull reconstruction in the Exocad—digital working model with 104 highlighted. **(E)** Right buccal view of the cranial reconstruction in the Exocad software—digital working model. **(F)** 104 extracted. **(G)** Final metallo-ceramic prosthodontic reconstruction at 104.

Using the aforementioned CAD/CAM techniques, a 1:1 copy of the 104 was constructed. The final restoration had the same length, width, and colour as the original 104 and could be inserted into its place in the maxilla. The design, titanium core, ceramic application, and comparison between the extracted tooth and ceramic core are shown in Figure 9.

## 4 Discussion

This is the first publication regarding an extensive multimodal investigation of an intravitam pink-discoloured tooth in a jaguar. This is also the first documentation of CAD/CAM reconstruction of a jaguar tooth.

Intravitam pink discolouration of the crown in a jaguar was solely reported by Schneider et al. (3) in one maxillary canine tooth that had no radiographic signs of endodontic disease. Similarly, in our case, CT images demonstrated no evidence of endodontic disease. According to Roux et al. (15), who examined 74 skulls of wild cats, pink discolouration was considered to have occurred post-mortem. Post-mortem pink teeth are well documented in the forensic literature (21–24) and are facilitated by skull preparations. Many cases of intravitam pink teeth remain unreported. For example, by analysing images in the articles published by Emily (19) and Almansa Ruiz et al. (13), intravitam pink teeth were visible in the images but were not reported by the authors. Anecdotally, many wildlife photographs also suggest underreporting of pink canine teeth in jaguars, leaving the subject open for further investigation.

A differential diagnosis should be established first with post-mortem pink teeth. In forensic human medicine, they typically appear because of environmental conditions, such as prolonged water immersion, when the head is in a position lower than the rest of the body, or as a result of asphyxia. According to Borrman et al. (22), these post-mortem pink teeth do not appear until 2 weeks after the person’s death, and Hartomo et al. (25) reported that they are rarely found in less than 4 weeks after the death of a human. In comparison, post-mortem phenomena in dogs and cats are described after thawing, with a duration of 10 to 21 days, and following refrigeration, between 28 and 30 days (26). According to Mehediratta et al. (27), 144 h post-mortem, dental pulp is completely decomposed in porcine teeth. The pulp tissues are among the most susceptible to decomposing immediately after death in humans (28), and the most important criterion for declaring post-mortem pink teeth is the presence of thrombi. Because the pulp tissues in the current study were intact, correlating with the keeper’s statement, we can conclude that the hypothesis of pink discolouration as a post-mortem change is invalidated.

Dental discolourations that occur during the lifetime of a patient may have various aetiologies. In humans and dogs, this may be due to a local cause or to a systemic cause with oral manifestations (29). According to Boy et al. (29), the local causes of intrinsic colouration in dogs include pulp necrosis, pulpal haemorrhage, and pulp tissue remnants after endodontic treatment. Remaining endodontic materials in the pulp chamber modify the colour of teeth over time in humans (30). From these possibilities, the one that best fits this case is pulpal haemorrhage, confirmed by the presence of antemortem thrombi and the fluorescence imaging results.

Systemic causes of dental discolouration include drug-related discolouration, such as tetracycline staining in humans (31, 32) and dogs (33). As the husbandry of the investigated jaguar was unknown, it cannot be stated whether tetracycline treatment occurred and could have contributed to the discolouration. However, tetracycline staining as a differential diagnosis was excluded because more teeth should be involved, and linear localization of the staining would be present in such a condition (31–33).

Fluorosis is a metabolic condition that alters the colour of human teeth by affecting enamel formation during amelogenesis (34). Clinically, it presents as opaque or chalky lines and patches on the tooth surface. The condition in humans typically arises from excessive fluoride intake, either through drinking water or toothpaste ingestion; however, this cause was not applicable in our case.

Several genetic disorders recognized for modifying tooth colour include congenital erythropoietic porphyria, dentinogenesis imperfecta, and dentin dysplasia (35–45). Congenital erythropoietic porphyria in cats (35) is a group of inherited disorders involving reduced activity of enzymes in the haem biosynthetic pathway. Porphyrin deposition, which produces discolouration, occurs only in actively mineralizing tissues. Reported cases include cattle, pigs, sheep, cats, dogs, mice, and rats (36–43). This condition is also generalized and not localized to a specific tooth. Another hereditary condition, Raine syndrome, is an autosomal recessive disorder characterized by hypomineralized teeth and diffuse discolouration. It has been described in both humans and dogs, with onset immediately after tooth eruption and consistently involves all teeth (44). Dentinogenesis imperfecta and dentin dysplasia (45) are other genetic conditions that cause amber tooth discolourations, as reported in dogs. Again, as genetic disorders, these processes involve all the teeth and thus may be excluded from our differential diagnosis in this case (45).

Our study supports the appearance of malocclusion among jaguars, which is reported in a very high percentage in Sao Paolo (47.61%) (17). This appearance is suspected to be related to genetic inbreeding among specimens kept in captivity. As the jaguar in this case did not present a precise family history record, genetic inbreeding could not be excluded. In all reported cases of jaguar malocclusion, the abnormality did not cause any alteration in the feeding process (17). Occlusal edge contact between incisor teeth leads to attrition and enamel loss. Tooth wear is one of the most common dental pathologies among carnivores, with a high prevalence of up to 80%, which is supported by the current case (3). In humans (46), dogs, and cats (47), the relationship between malocclusions and further complications over the lifetime of the individual is well established. This finding supports the importance of understanding the prevalence and determining the factors contributing to malocclusions. This finding and establishing the need for further orthodontic treatment in jaguars are topics to be evaluated in the future.

Uncomplicated crown fractures, in this case present at tooth 103, are reported in a lower percentage, between 7.1% (3) in captive jaguars and 26.8% (48) in captive neotropical wild carnivores, including the ocelot (*Leopardus pardalis*), cougar (*Puma concolor*), jaguarundi (*Puma yagouaroundi*), margay (*Leopardus wiedii*), pampas cat (*Leopardus colocolo*), crab-eating fox (*Cerdocyon thous*), hoary fox (*Pseudalopex vetulus*), and maned wolf (*Chrysocyon brachyurus*). Complicated crown fractures are more frequently encountered because of the biting force developed by durophagy and the killing techniques used on prey (4–9). In this case, the first right mandibular incisor tooth was clinically absent from the dental arch, with a retained root found on the CT scan. More than 50% of the population of captive jaguars have been reported to have absent teeth, at 53.3% (3) or 67.7% (30), with incisor teeth being most commonly absent at 70.4% (3).

Studies have reported a prevalence of 38% (14) and 58% (48) for calculus in adult jaguars. Regarding periodontal examination, no signs of periodontal disease were confirmed clinically or by CT imaging. The authors support the findings of Schneider et al. (3) that a greater than 3 mm pocket depth is pathological when analysing the oral cavity of a jaguar.

Pulpal cavities, as visualized on CT images, were in concordance with the jaguar’s age compared to tooth ageing in domestic cats, dogs, and coyotes (49–52). Secondary dentin deposition is correlated with ageing in that pulp cavity sizes become progressively smaller with age if the tooth does not suffer any trauma that would result in a non-vital pulp and block normal development (49–52). In the analysed canine tooth, the pulp cavity had a very small width, which the authors considered to be correlated with 12 years of life. Further support for this conclusion is that all four canine teeth were pink and discoloured, with similar pulp cavity widths, and no periapical lesions.

Yellow discolouration of the canine teeth due to ageing in wild cats, specifically the Eurasian lynx (*Lynx lynx*), was a subject addressed by Marti et al. (53). In this study, the authors considered this discolouration to be physiological in the Eurasian Lynx without investigating it further microscopically or radiologically. For age approximation in wild cats, it may be a helpful tool, but without correlating the colour to further histological and radiological findings, its cause remains open to discussion and is not applicable in this study.

Extraction of the 104 with piezoelectric surgery was chosen based on the authors’ clinical experience and the objective of preserving the bone and lowering the risk of damaging the thin vestibular cortical bone (54).

Regarding the microscopic findings in GT-stained histological specimens, the literature described that odontoblast and cell-free zones are missing from the sections in the present study (55). The presence of the cell-free zone as part of the dental pulp in non-human mammals is open to discussion (56). Equine teeth (57) and rat teeth (58) lack this zone, while human teeth and non-human primate teeth (56) do have it. Nedelea et al. (59) reported the presence of a reduced cell-free zone in dogs. While Schneider et al. (3) presented histologic photomicrographs of jaguars’ teeth, they do not refer to the three/four zones of the dental pulp, instead reporting only on the histopathologic abnormalities found.

The cell-rich zone and central part of the coronal pulp in this study had a higher degree of vascularization compared to domestic cats’ normal pulp tissue (60). In domestic cats, there is a uniform distribution of the vessels, but in the present case, the distribution is uneven. Compared to the results reported by Vongsavan and Matthews (60), who reported that only 42.9% of the dental pulp area is occupied by vessels in the core of the pulp, the described intravitam pink tooth had a higher percentage of pulp area representing blood vessels, with over 50% of the blood vessels presenting thrombi with a high percentage of canalization. This 50% number was obtained by visually counting the vessels, categorized as small, medium, and large, as well as those with thrombi, on five stained sections (for example, in the section shown in the figure, the authors counted approximately 114 vessels, 68 of which had thrombi). As it is a visual sampling method and not a precise one, the authors approximated the percentage. The thrombi canalization process, also known as recanalization (61), is a chronic process (62) studied mainly in human medicine, and it was initially reported in 1973 by Sevitt (63, 64). It is a remodelling process to reopen the vessel’s lumen to restore blood flow. This process differs from the vascularization process in veins, where the new capillaries are formed within the thrombus and communicate with *vasa venarum* (63). While Sevitt reported (64) that the anchoring zone of the thrombi is usually one-sixth to one-third of the vein circumference, in this case, anchoring of the thrombi was observed to occur along half to three-quarters, or sometimes even the entire circumference of a given vessel. Concerning the structural features of the detected thrombi, in the present case, the occluding thrombi included fibrinous material along with trapped fading blood cells. In the case of the recanalization of thrombi, discrete areas of fibrous organization and scattered siderophages were identified throughout the thrombotic mass, indicating a chronic process (63). Although it was initially believed that recanalization occurs 6 months to years after thrombus formation, it is now known that the recanalization process begins in the first week of thrombus formation (61). According to Carrasco et al. (28), well-maintained preservation of blood vessel cells is still noticeable within the first 24 h after death. Therefore, the presented tooth details are representative of an intravitam pink tooth.

According to Feigin et al. (65), the majority of stained canine teeth in dogs were non-vital (87.6%), with approximately two-thirds of these cases showing no histological signs of endodontic or periodontal inflammation. More than half of the intrinsically stained teeth also lacked evidence of coronal injury. Radiographic evidence of endodontic disease was present in 57% of the intrinsically stained teeth, radiographic evidence of periodontal disease was present in 48, and 28% had radiographic evidence of tooth resorption. In contrast, the teeth examined in our study histologically showed no necrosis or inflammation, as indicated by the absence of leukocytes, necrotic debris, or haemorrhage. Instead, the presence of recanalized blood vessels supported the interpretation of pulp vitality. Likewise, computed tomography revealed no pathological changes, such as arrested root maturation or indicators of endodontic or periodontal disease. Although Feigin et al. noted that teeth with normal radiographic findings can still be non-vital, our histological observations of intact vessels and the absence of inflammatory infiltrates strongly support the vitality in this case. Accordingly, their conclusions may also apply to jaguars’ pink-stained teeth: discolouration without histological evidence of inflammation can result from causes other than endodontic or periodontal disease, such as trauma-induced pulpal haemorrhage. In this context, trauma resulting from malocclusion or attrition should also be considered a potential etiological factor in the present case.

Haemoglobin’s fluorescence with a 470 nm LED for excitation and a 515 nm long-pass emission filter has been previously reported by Peng and Liu (66). Our analysis illustrated fluorescence in the thrombi and dentin. Thus, the pink appearance of canine teeth is due to the presence of haemoglobin/haemoglobin products in the dentin.

Regarding the TEM findings, unlike human dentin, where the peritubular dentin is demineralized (67), in the analysed specimen, the degree of mineralization was the same, showing no difference between intertubular and peritubular dentin. Several odontoblastic processes in the dentinal tubules showed a multifocal vacuolated appearance. In most circumstances, a space between odontoblastic processes and the dentinal tubules was observed. However, in our study, the dimensions of the dentinal tubules appeared larger than those reported by DeLaurier et al. (68), and compared to our findings, smaller spaces were reported by others between the dentinal tubules. As for the structure of dentin, a 3D orientation of the collagen fibres was identified in both dentinal zones, with a lower collagen fibre density in the peritubular region compared to the intertubular dentinal zone. The last zone displayed a higher collagen fibre density organized in interlacing bundles with a banding pattern. Taken together, the TEM analysis provides important insights into the ultrastructural characteristics of dentin in the examined specimen. Unlike human dentin, where peritubular regions are typically demineralized (67), no differences in the degree of mineralization were observed between the intertubular and peritubular dentin in the present case. This finding highlights a fundamental divergence in tissue organization that may reflect species-specific variations in mineral metabolism and dentinogenesis. The observation of vacuolated odontoblastic processes and the consistent presence of a space between odontoblastic processes and dentinal tubules further support the notion of ultrastructural heterogeneity within this tissue. Notably, the tubule dimensions were larger than those previously reported by DeLaurier et al. (68), suggesting potential functional or adaptive modifications related to dentinal permeability and sensitivity. Regarding the collagen matrix, the identification of a three-dimensional orientation of fibres in both dentinal zones, combined with differences in fibre density (lower collagen fibre density in the peritubular region compared to the intertubular dentinal zone), emphasizes a highly specialized microarchitecture. The lower collagen density in the peritubular region, in contrast to the densely interlaced bundles of the intertubular zone displaying a characteristic banding pattern, suggests distinct structural roles that contribute to the overall biomechanical properties of the dentin. By demonstrating the structural and organizational features that differ from those classically described in humans, this study contributes to a broader understanding of dentin biology and provides a valuable reference point for future comparative and functional investigations in jaguars.

Computer-aided design (CAD) and computer-aided manufacturing (CAM) dental reconstructions have become very popular in modern dentistry, and a branch known as digital dentistry has developed since it was first introduced in 1980 (69). Since then, every specialty in human dentistry has introduced CAD/CAM in daily practice. CAD/CAM is used in veterinary dentistry and oral surgery of small animals to create prosthodontic metallic crowns for dogs (70), printed models of previous oncologic surgeries (71), or custom-made titanium plates to reconstruct the surgical defects resulting from tumour ablations (72). Using a CT scan, the dental laboratory can obtain a digital model that can be printed or used directly in the application. In this case, the extracted canine tooth was CAD, and the tooth’s core was printed. Human teeth have smaller dimensions than the canine teeth of a jaguar, and the printing machine was designed for human dentistry. The length of canine teeth in humans varies between 16 mm and 28 mm (73). Thus, the 75 mm length of the extracted jaguar canine tooth had to be divided into three parts. It was necessary to make the core from three parts that were interlocked, like puzzle pieces. Subsequently, the ceramic layers were applied to achieve the required colour, and unlike in human prosthodontics, pink shades, which are usually used for the gingival part of the prosthodontic pieces, had to be combined for a satisfactory result.

One possible explanation for the observed pink discolouration could be that a blood circulation disorder occurred in the dental pulp at some point during the jaguar’s lifetime. Canine teeth play an essential role in durophagy and the killing method; therefore, a high mechanical pressure might have caused compression or damage to the vascular bundle. Consequently, a disparity between blood supply and venous drainage could have occurred, causing entrapment of a quantity of blood and favouring thrombus formation along with haemorrhage by diapedesis. As drainage could have been inadequate, haemolysis might have led to the appearance of free haemoglobin and haemoglobin products. Haemoglobin’s dimensions are on the order of nanometres (74), while erythrocytes vary in the feline group between 5 and 6 μm in domestic cats (75) to 7.5 μm in tigers (76). Comparing these dimensions to the dentinal tubular dimensions, we suspect that the haemoglobin derivatives invaded the dentinal tubules and produced a pink colour. In conclusion, our study proposes a mechanism for intravitam pink-discoloured maxillary canine teeth in a jaguar.

### 4.1 Limitations

This study presents a valuable single-case analysis, although no unaffected teeth were available for comparison. Inclusion of an unaffected tooth in the extraction and analysis would have provided additional insights. The authors compared their findings with those of other species. However, an examination of the surrounding bone, in addition to the tooth itself, could have yielded further information regarding periodontal issues and the condition of the alveolar bone. Limited knowledge of the animal’s history—including whether it was wild-born or captive, its diet, and overall medical background—represents an additional constraint. Antemortem observations were not documented and relied solely on the keeper’s statement, which is another limitation. Future studies investigating the microscopic appearance of unaffected and other pink-discoloured teeth are warranted to determine whether this represents a pathological condition and whether medical attention is required. Until such investigations clarify the nature and implications of intravitam pink teeth in jaguars, particularly regarding oral homeostasis and feeding processes, the topic remains open to further research.

## Data Availability

The raw data supporting the conclusions of this article will be made available by the authors, without undue reservation.
